# Vardenafil increases intracellular accumulation of the most prevalent mutant cystic fibrosis transmembrane conductance regulator (CTFR) in human bronchial epithelial cells

**DOI:** 10.1242/bio.053116

**Published:** 2020-08-25

**Authors:** Barbara Dhooghe, Caroline Bouzin, Angélique Mottais, Emmanuel Hermans, Martial Delion, Nadtha Panin, Sabrina Noel, Teresinha Leal

**Affiliations:** 1 Louvain Centre for Toxicology and Applied Pharmacology, Institut de Recherche Expérimentale et Clinique, Université Catholique de Louvain, 1200, Brussels, Belgium; 2 Institut de Recherche Expérimentale et Clinique, Cell Imaging Platform, Université Catholique de Louvain, 1200, Brussels, Belgium; 3 Institute of Neurosciences, Faculté de Pharmacie et Sciences Biomédicales, Université Catholique de Louvain, 1200, Brussels, Belgium

**Keywords:** Cystic Fibrosis, CFTR, Vardenafil, Phosphodiesterase type 5 inhibitor, cGMP, PKG

## Abstract

Cystic fibrosis (CF) is a genetic disease characterized by progressive lung and chronic digestive manifestations. We have shown that therapeutic doses of vardenafil, a phosphodiesterase type 5 (PDE5) inhibitor, corrects CF Transmembrane conductance Regulator (CFTR)-dependent chloride transport in respiratory and intestinal tissues of F508del homozygous mice. Here, we studied the effect of vardenafil on CFTR in 16HBE14o^–^ and CFBE41o^−^ cell lines. First, the expression levels of PDE5 mRNA in these cell lines were monitored. The two cell lines were exposed to different drugs (dimethyl sulfoxide, 8-Br-cGMP, forskolin or vardenafil). The cAMP and cGMP intracellular concentrations were measured. Finally, we localised the CFTR by immunolabelling. PDE5 was similarly expressed in both wild-type and in CF cells. A fast and transient rise in cGMP intracellular contents followed treatment with vardenafil, confirming its PDE5 inhibitory effect. We showed that vardenafil promoted both the early steps of the cellular processing and the trafficking of F508del without fully addressing the protein to the plasma membrane. The effect was not reproduced by the brominated cGMP analogue and it was not prevented by the combination of a protein kinase G (PKG) inhibitor and vardenafil. These findings support the view that vardenafil partially rescues F508del through cGMP/PKG-independent mechanisms.

## Abstract (precis)

**Summary:** Our findings support the view that vardenafil, a phosphodiesterase type 5 inhibitor, partially rescues F508del protein through cGMP/PKG-independent mechanisms in cystic fibrosis cell line.

## INTRODUCTION

Despite remarkable progress achieved in the understanding of its pathophysiology during the past two decades, cystic fibrosis (CF) remains a life-threatening inherited disorder. Its most conspicuous feature is a respiratory phenotype characterised by chronic airway infection and inflammation, mucus-obstructed airways and progressive bronchiectasis, finally leading to respiratory failure. Furthermore, almost 90% of patients develop pancreatic insufficiency that, together with intestinal manifestations, lead to steatorrhea and failure to thrive. The disease is caused by mutations in the *CF Transmembrane conductance Regulator* (*CFTR*) gene (Riordan et al., 1989) coding for an ATP-binding cassette (ABC) protein (Dean et al., 2001) mainly functioning as a chloride channel in apical membranes of epithelial cells. CFTR is an integral glycoprotein comprised of two membrane-spanning domains (MSD), two nucleotide-binding domains (NBD) and a regulatory (R) domain. The R domain, unique to CFTR, is rich in consensus sites for protein kinase A (PKA) phosphorylation (Chang et al., 1993). The most frequent CFTR mutation, F508del, corresponding to deletion of phenylalanine 508 in NBD1, results in misfolding and mistrafficking of the protein, with its retention in the endoplasmic reticulum (ER) and premature proteasomal degradation (Cheng et al., 1990). Although some mutant protein escapes the ER and reaches apical membranes, it exhibits a gating defect with reduced channel opening, a stability defect and an increased endocytic turnover in plasma membranes (Lukacs et al., 1993); these defects impact on fluid and electrolyte transport in exocrine epithelia.

The clinical manifestations due to loss of CFTR function have long been managed with supportive care only. In the past decade, a number of compounds have been identified as pharmacological modulators of CFTR and some have been tested in clinical trials (De Boeck and Amaral, 2016; Dhooghe et al., 2016). Clinical and basic studies have unanimously brought up the concept that combining drugs relying on different mechanisms of action is necessary to counteract the multiple defects of F508del-CFTR (Wang et al., 2007; Thibodeau et al., 2010; Farinha et al., 2013; Boinot et al., 2014). Two correctors, lumacaftor and tezacaftor, targeting the misprocessing and trafficking defects caused by the mutation, have been approved for treatment, alone (Clancy et al., 2012) or in combination (Boyle et al., 2014; Taylor-Cousar et al., 2017; Wainwright et al., 2015) with the potentiator ivacaftor. Ivacaftor increases the channel-open probability (i.e. the fraction of time that a single CFTR protein channel is open and transporting ions) of normal and mutant CFTR protein. Significant improvements in pulmonary function, body weight and CFTR transport activity have been demonstrated with ivacaftor in a broad range of *CFTR* mutations with gating defects and of other mutations that result in some CFTR protein expressed at the epithelial cell surface (Davies et al., 2013; De Boeck et al., 2014). However, combinations of lumacaftor and ivacaftor for F508del mutation have shown only modest clinical benefits in lung function and nutritional status, and in reduced frequency of exacerbations (Wainwright et al., 2015). Therefore, basic therapeutic strategies aiming at rescuing mistrafficking and function of the most common and one of the most severe *CFTR* mutations are still crucially needed. Recently, a triple combination therapy including elexacaftor, a next-generation corrector, and tezacaftor and ivacaftor, has resulted in improved protein function in patients with one or two F508del alleles (Keating et al., 2018).

A well-characterised signalling pathway regulating CFTR activity relies on intracellular cyclic adenosine monophosphate (cAMP) through PKA-dependent phosphorylation of the R domain (Chang et al., 1993). Evidence supports cGMP-dependent protein kinase G (PKG) as another regulator of CFTR phosphorylation and activity. Based on its cytosolic localisation, involvement of the isoform I of PKG (PKGI) in modulating CFTR phosphorylation has been discarded. Studies have shown that consensus sites for PKA in the R domain could be activated and phosphorylated by isoform II of PKG (PKGII) in excised membrane patches from NIH-3T3 fibroblasts and from a rat intestinal cell line (IEC-CF7), suggesting that PKGII phosphorylates CFTR at sites overlapping those phosphorylated by PKA (French et al., 1995). The fact that PKGII contains a consensus N-terminal myristoylation sequence, targeting it to a membrane location, supports the assumption that it may phosphorylate CFTR, also an integral membrane protein (Vaandrager et al., 1996, 1998). It has also been shown that cGMP stimulates CFTR expression in the surface of villus enterocytes in rats in a PKGII-dependent way (Golin-Bisello et al., 2005), thus supporting the idea that modulation of the cGMP pathway could be a potential strategy to rescue F508del-CFTR mistrafficking.

Inhibiting the breakdown of cGMP is a well-known approach to modulate cGMP signalling. Vardenafil, sildenafil and tadalafil, clinically approved drugs for the treatment of erectile dysfunction (Corbin, 2004) and pulmonary arterial hypertension (Hemnes and Champion, 2006), are highly selective inhibitors of cGMP-specific phosphodiesterase type 5 (PDE5). High-throughput screening strategies have identified sildenafil as a potential compound able to rescue F508del-CFTR (Carlile et al., 2007). Cell-based studies have shown that supratherapeutic doses of sildenafil were able to correct the localisation of F508del-CFTR protein in nasal epithelial cells harvested from patients with CF (Dormer et al., 2005). We have shown that intraperitoneal or inhaled therapeutic doses of PDE5 inhibitors corrected CFTR-dependent chloride transport in nasal (Lubamba et al., 2008, 2011) and rectal (Dhooghe et al., 2013) mucosae of F508del-CF homozygous mice. Vardenafil promotes F508del-CFTR accumulation and redistribution towards the membrane region of colonocytes from F508del-CF mice, indicating that the drug acts both as a corrector and as a potentiator of CFTR, thus making it a potential candidate for CF therapy (Dhooghe et al., 2013). Vardenafil is a more potent and longer-acting cGMP accumulator than sildenafil (Gresser and Gleiter, 2002). In addition, it displays anti-inflammatory properties in acutely induced airway inflammation in CF (Lubamba et al., 2012) and it modulates a pro-inflammatory and pro-fibrogenic phenotype in CF fibroblasts (Huaux et al., 2013). The lowest concentration to combine correcting effects on transepithelial ion transport (Dhooghe et al., 2013; Lubamba et al., 2008, 2011) and on inflammatory/fibrogenic (Huaux et al., 2013; Lubamba et al., 2012) responses in CF was 10 μM vardenafil.

As the effect of vardenafil on CFTR function has been previously evidenced using a mouse model of the disease (Dhooghe et al., 2013; Huaux et al., 2013; Lubamba et al., 2008, 2011, 2012), this work was designed to investigate the impact of vardenafil on cellular cGMP accumulation and CFTR localisation in human-derived cell-based airway epithelium models grown on an impermeable support (Gruenert et al., 2004; Ehrhardt et al., 2006). We confirmed that vardenafil promotes a rapid and transient increase in cellular cGMP contents and that it promotes early and intermediate steps of the intracellular processing and trafficking of F508del-CFTR in bronchial epithelial cells without fully addressing the protein to the plasma membrane.

## RESULTS

### Wild-type and CF human bronchial epithelial cells express PDE5A

**Fig. 1.**
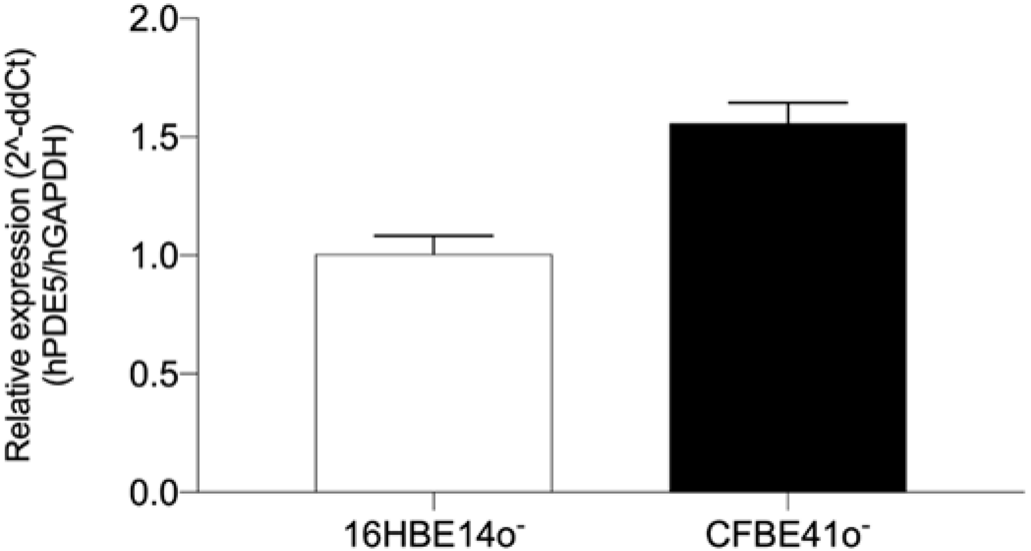
**Quantification of human PDE5A (hPDE5) mRNA in 16HBE14o^–^ cells and CFBE41o^–^.** Relative expression by RT-qPCR normalised to human GAPDH (hGAPDH) mRNA. Data expressed as mean±s.e.m. (*n*=7). As the relative quantification was lower than 2 in the CFBE41o-group, no significantly different expression of hPDE5 was revealed between the two cell lines.

The PDE5 enzyme, encoded by the *PDE5A* gene, is expressed in many tissues including the lungs of mammals (Francis and Corbin, 1988; Thomas et al., 1990; Lin, 2004). The immortalised parental CF human bronchial epithelial cell line (CFBE41o^−^) and the corresponding wild-type cell line (16HBE14o^−^) were kindly given by P. B. Davis (Case Western Reserve University, Cleveland, Ohio, USA). They were originally generated by Dieter C. Gruenert (California Pacific Medical Center Research Institute, San Francisco, CA, USA), from the first bifurcation of a human bronchus (Gruenert et al., 2004; Ehrhardt et al., 2006). The 16HBE14o^–^ cells express endogenous wild-type CFTR and the CFBE41o^–^ is homozygous for the F508del CFTR mutation. They are valuable cell-based models to test the potentiality of small molecules for CF pharmacotherapy (Illek et al., 2008). To validate 16HBE14o^−^ and CFBE41o^−^ cells as tools to monitor PDE5 inhibition by vardenafil, we assessed the endogenous expression of PDE5A mRNA by RT-qPCR (normalised to the expression of GAPDH mRNA). We found that PDE5A mRNA is equally expressed in both cell lines, irrespective of the genotype (Fig. 1). These data highlight the fact that human bronchial epithelial cells endogenously express PDE5, justifying the use of PDE5 inhibitors to target enzyme activity in these cells.

### Vardenafil provokes a fast and transient rise of intracellular cGMP concentrations

**Fig. 2.**
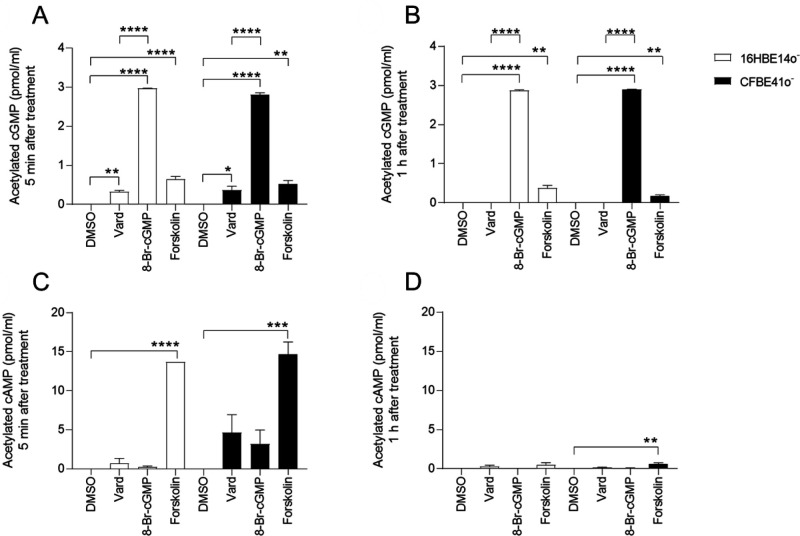
**Quantification of acetylated cGMP and cAMP intracellular contents in 16HBE14o^–^ and CFBE41o^–^ cells.** cGMP (A,B) and cAMP (C,D) concentrations 5 min or 1 h after treatment with either DMSO, 10 µM vardenafil, 10 µM 8-Br-cGMP or 10 µM forskolin. 16HBE14o^–^ (white columns) and CFBE41o^–^ (black columns). Data expressed as mean±s.e.m. (triplicate of three individual samples). To minimise inter-assay variability, data from different assays were not pooled and intra-assay comparisons were made within each of three independent experiments performed for confirmation. Asterisks indicate levels of significance of between-group comparisons performed by ANOVA with post-hoc analysis made by using Student's *t*-test or the Tukey–Kramer HSD test, as adequate (*P<0.05; **P<0.01; ***P<0.001; ****P<0.0001).

To confirm the effect of vardenafil as a PDE5 inhibitor in 16HBE14o^−^ and CFBE41o^−^ cells, we monitored cGMP concentrations after treatment with 10 µM vardenafil in cell lysates obtained from cultures of the cells. DMSO, used to solubilize vardenafil, served as a negative control after verifying that it did not induce significant changes in the analyses performed in wild-type or CF cells (Fig. 2). In parallel, the effects of the brominated analogue 8-Br-cGMP and that of the adenylate cyclase activator forskolin were used as positive controls of cGMP and cAMP monitoring.

As expected, treatment with 10 µM of the cell-permeant cGMP analogue 8-Br-cGMP increased intracellular cGMP contents both in the wild-type and in the CF cell lines (Fig. 2A). The effect lasted at least 1 h (Fig. 2B), in line with a high resistance of the cGMP analogue to hydrolysis by PDEs. As also expected, treatment of both cell types with forskolin was followed by a rapid rise in intracellular cAMP contents, irrespective of the genotype (Fig. 2C). A significant effect of forskolin on cAMP was still detected after 1 h in CF cells, though at a much lower level (Fig. 2D).

Vardenafil induced a fast rise in cGMP intracellular contents in both 16HBE14o^−^ and in CFBE41o^−^ cell lines. As a matter of fact, after 5 min of vardenafil treatment, cGMP concentrations were larger than those found in control conditions (Fig. 2A). The increase did not persist 1 h after treatment (Fig. 2B), suggesting that the inhibitory effect of vardenafil on PDE5 activity could be rapidly counterbalanced by effective downregulation of cGMP production by guanylate cyclases. The magnitude of the effect was similar for concentrations ranging from 1 to 100 µM vardenafil, either 5 min or 1 h after treatment (Fig. S1). These findings indicate that the maximal inhibitory effect on PDE5 in bronchial epithelial cells was obtained with any tested concentration, no further increase of cGMP intracellular contents being observed with larger supratherapeutic vardenafil concentrations (30–100 µM). The 10 μM vardenafil concentration was selected based on its potential clinical relevance as the lowest concentration to combine the correcting effects on transepithelial ion transport (Dhooghe et al., 2013; Lubamba et al., 2011) and on inflammatory/fibrogenic responses (Huaux et al., 2013; Ehrhardt et al., 2006).

### No directional crosstalk from cGMP to cAMP

To test the presence of bi-directional crosstalk between cGMP and cAMP pathways (Zaccolo and Movsesian, 2007), we also investigated the effect of vardenafil or of 8-Br-cGMP on cAMP concentrations and that of forskolin on cGMP concentrations. Neither in wild-type nor in CF cells was any significant increase in cAMP concentrations observed after treatment with vardenafil nor with the cGMP analogue, compared to the levels found after DMSO (Fig. 2C,D). In CF cells, the *P*-value for the comparison between DMSO and vardenafil was 0.0803 and that between DMSO and 8-Br-cGMP was 0.2545.

In both wild-type and in CF cells, a directional cAMP-to-cGMP crosstalk, i.e. forskolin-triggered cGMP response, was demonstrated by an increase in cGMP concentrations following forskolin treatment (Fig. 2A,B). The effect was apparently not related to the presence of the F508del mutation as it was observed at similar levels in both CF and in wild-type cells. Previously published data have shown that guanylate cyclase is also stimulated by forskolin (Brandi et al., 1984; Ho et al., 1989). A unidirectional crosstalk between the two pathways was shown: the cAMP-to-cGMP crosstalk was fast, long lasting and unrelated to the genotype; the cGMP-to-cAMP crosstalk, i.e. 8-Br-cGMP-triggered cAMP response, was absent in both normal and CF cells and was not influenced by vardenafil (Fig. 2C,D).

### Vardenafil increases F508del-CFTR expression independently of the cGMP/PKG-signalling pathway

**Fig. 3.**
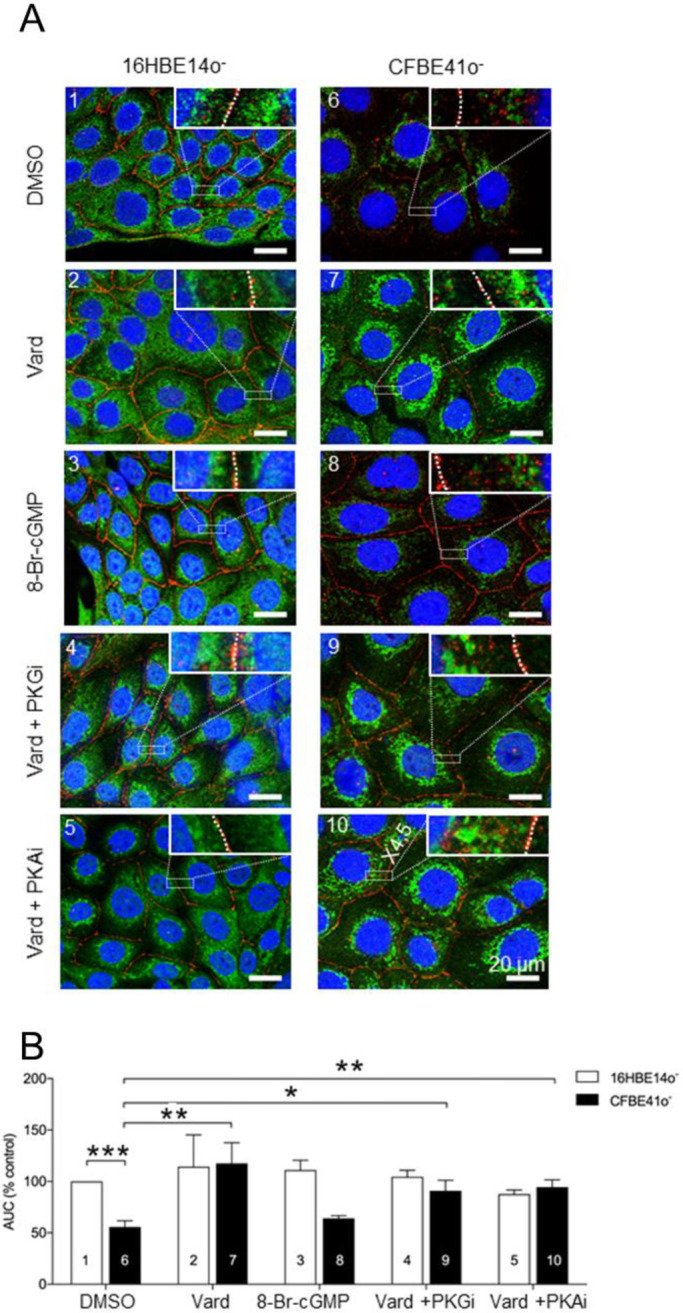
**Representative immunohistochemical images of CFTR in 16HBE14o^–^ and in CFBE41o^–^ cells.** (A) Labelling of CFTR (green) and zonula occludens (Z0-1, red) 1 h after incubation with DMSO (A1 and A6), 10 µM 8-Br-cGMP (A3 and A8), 10 µM vardenafil alone (A2 and A7) or in combination with 1 µM PKA inhibitor (A5 and A10) or 1 µM PKG inhibitor (A4 and A9) in wild-type 16HBE14o^−^ (left panel; 1–5) and in CFBE41o^−^ cells (right panel; 6–10). In each picture, a small rectangle containing a region extending from the plasma membrane to the nucleus is enlarged (×4.5) in insert. Nuclei (blue) stained by DAPI. (B) Average area under the curves (AUC) obtained from the intensity of the CFTR signals quantified by morphometric analysis of cross-sectional profiles of individual cells (*n*=9) and expressed as a measure of total CFTR protein expression (nuclei excluded). Each condition has been normalised to that measured in the control group (DMSO-treated 16HBE14o^−^ cells). Data are expressed as mean±s.e.m. Asterisks indicate levels of significance of between-group comparisons performed by ANOVA with post-hoc analysis made by using Student's *t*-test or Tukey–Kramer HSD test, as appropriate (*P<0.05; **P<0.01; ***P<0.001).

Next, we investigated, by immunostaining studies, the effect of the drug alone or in combination with a PKG inhibitor (Rp-8-Br-PET-cGMPS) or a PKA inhibitor (H-89) on CFTR localisation. Both PK inhibitors are membrane permeant, metabolically stable, very potent and highly selective: the Ki of Rp-8-Br-PET-cGMPS is about 35 nM for PKG while the value for PKA is at least 300-fold higher (Schwede et al., 2000). Conversely, the Ki of H89 for PKA is very low (48 nM) and even at a 200-fold higher concentration (10 μM), it does not inhibit PKG (Lochner and Moolman, 2006). The experiments were performed in parallel with the brominated cGMP analogue. In control conditions, the CFTR fluorescence signal was distributed throughout the cytoplasm of 16HBE14o^−^ cells up to the juxta-membrane areas (Fig. 3A1), while in CFBE41o^−^ cells, the total intensity of the signal was decreased and it was mostly detected in the juxta-nuclear areas (Fig. 3A6), enlightening the mistrafficking of F508del-CFTR protein. Treatment of CFBE41o^−^ cells for 1 h with 10 µM vardenafil was followed by an increase in total intensity of the CFTR fluorescence signal (Fig. 3A7) and it was not influenced by treatment with 8-Br-cGMP, (Fig. 3A8), suggesting that expression of F508del-CFTR and correction of its mistrafficking are not directly mediated by intracellular accumulation of cGMP. No significant effect of vardenafil (Fig. 3A2) or 8-Br-cGMP (Fig. 3A3) was noticed in 16HBE14o^−^ cells.

To better understand the effect of vardenafil on correction of F508del-CFTR, cells were concomitantly treated with the drug and with PKG or PKA inhibitors. Co-treatment with inhibitors of PKG (Fig. 3A9) or PKA (Fig. 3A10) did not influence the effect of vardenafil on F508del-CFTR cell accumulation. Similarly, no significant effect of the co-treatment with vardenafil and PKGi (Fig. 3A4) or PKAi (Fig. 3A5) was observed in 16HBE14o^–^ cells. The intensity of the CFTR fluorescence signal was indeed unchanged in CFBE41o^−^ and in 16HBE14o^–^ cells co-treated with vardenafil and the inhibitors of PK A or G. The *P*-value for the comparison between CF vardenafil (Fig. 3B7) and CF vardenafil together with PKGi (Fig. 3B9) is 0.699 and that for the comparison between CF vardenafil (Fig. 3B7) and CF vardenafil together with PKAi (Fig. 3B10) is 0.818.

To quantitatively substantiate the observations, we carried out comparative morphometric analyses of immunostained cells in different conditions (Fig. 3B1–B10). Using the AxioVision Software, pixel intensities of the CFTR signal were measured in the cytoplasmic part of cross-sectional scans drawn through the widest part of the nucleus of individual cells. The mean CFTR-related intensity across each cell (*n*=9 per condition, randomly selected, nuclei excluded) was taken as representative of the CFTR signal normalised to that measured in DMSO-treated 16HBE14o^−^ cells as a reference after verifying that treatment with DMSO does not interfere with the measured fluorescence intensity. The normalised area under the curve in DMSO-treated CFBE41o^−^ cells (Fig. 3B6) was reduced by almost half compared to DMSO-treated 16HBE14o^−^ cells (Fig. 3B1) and vardenafil increased it about twofold to levels similar to those found in controls (Fig. 3B7). These findings highlight the fact that vardenafil increased the detected amount of CFTR. The effect of vardenafil was not modified by co-treatment with a PKG (Fig. 3B9) or PKA (Fig. 3B10) inhibitor in CF cells, indicating that the increased CFTR intracellular accumulation upon vardenafil treatment is not related to the PK signalling cascades triggered by the cGMP increase.

### Vardenafil triggers F508del-CFTR cellular processing and promotes its accumulation in juxta-membrane areas

**Fig. 4.**
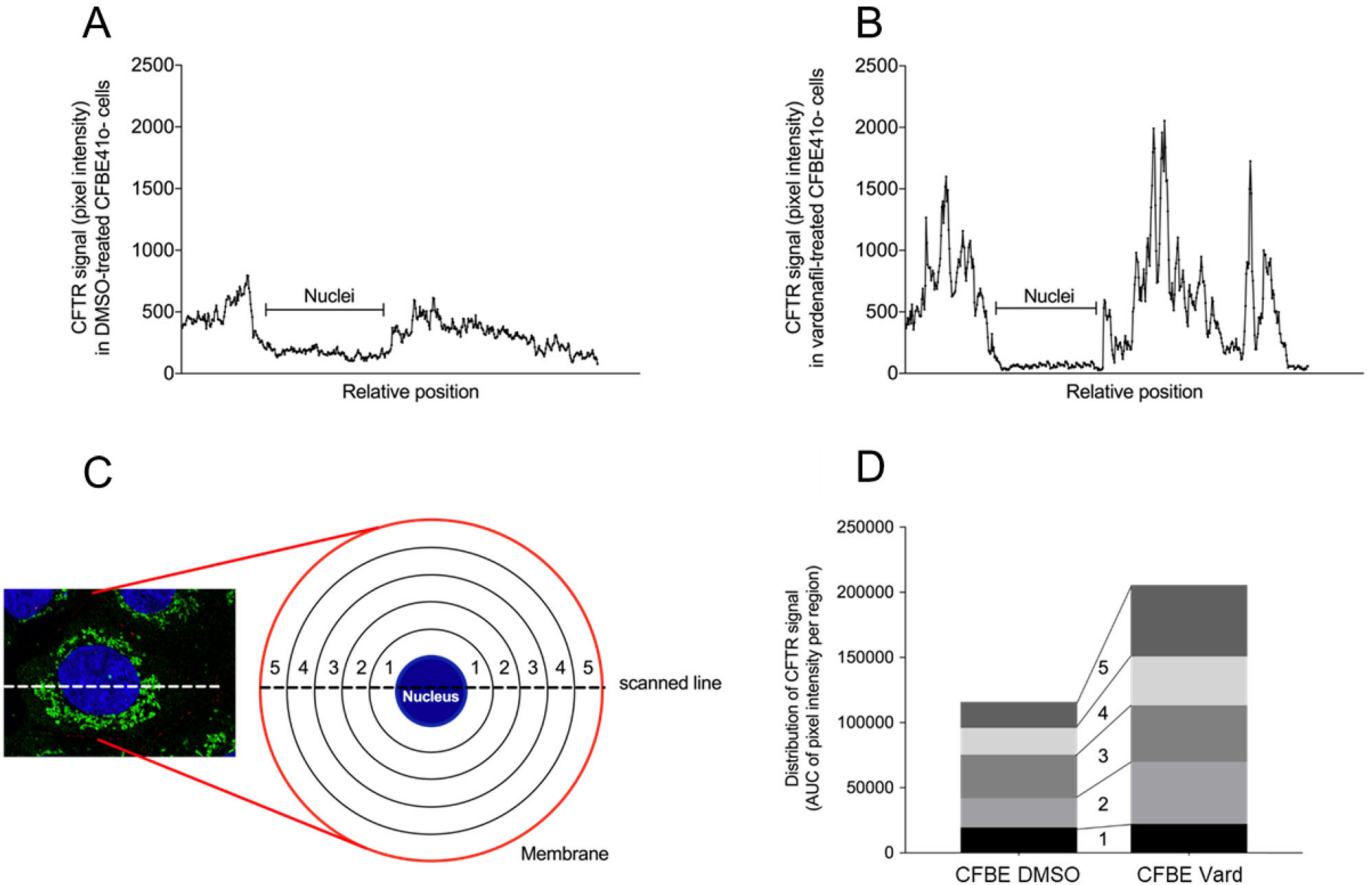
**Morphometric analysis of CFTR immunofluorescence of CFBE41o^–^ cells.** Distribution of the CFTR signal intensity quantified by morphometric analysis of cross-sectional profiles of individual cells (*n*=9) treated with either (A) DMSO or with (B) vardenafil. (C) Illustration of dividing a cell scan into five segments from the juxta-nuclear region (1) to the membrane region (5). (D) Effect of the vardenafil treatment on the distribution of the CFTR signal. *n*=9 for each condition.

To quantify the effect of vardenafil on the cellular distribution of F508del-CFTR, nine cells were selected at random in images of DMSO- and vardenafil-treated CF cells. Three independent experiments were performed for confirmation. Each cell was scanned along a line through the widest part of the nucleus. Morphometric measurements of the position of the CFTR signal were normalised at each radial extension of the cell individually, setting in each case the distance from the nuclear membrane to the plasma membrane as equal to 1, and expressing the position of the CFTR signal along the line as a corresponding fraction of 1. The scans of the nine cells of each of the two conditions were added and the results are given in Fig. 4A and B. In both cases, the region corresponding to the nucleus was identified. To analyse the distribution of the signal, the cytoplasmic part of the total scans of Fig. 4A and B were divided into five segments of equal lengths (Fig. 4C), the first segment corresponding to the juxta-nuclear region and the fifth segment to the membrane region. Areas under the curves were computed for each segment and the results are given in Fig. 4D. They show that in DMSO-treated 16HBE14o^–^ cells, the CFTR signal was distributed throughout the cytoplasm (Fig. S2) while in CFBE41o^–^ cells it was distributed in the five segments with only 15% of the signal being located within the fifth juxta-membrane segment (Fig. 4A,D). The distribution was modified by vardenafil treatment. While the CFTR signal remained unchanged in the juxta-nuclear (first segment) region, it was indeed almost tripled (increased 2.9-fold) in the fifth juxta-membrane segment, that alone contained about a quarter of the total signal of vardenafil-treated cells. However, the vardenafil treatment did not completely correct the distribution of the protein, a consistent fraction remaining in the intermediate segments. These results confirm the triggering effect of vardenafil on the processing and trafficking of F508del-CFTR, but without fully addressing the protein to the cell membrane.

## DISCUSSION

In this work, we provided novel data on the effect of vardenafil on intracellular accumulation of cGMP and localisation of CFTR in the airway epithelium with regard to a therapeutic potential for CF. We used human bronchial epithelial cell lines to study the impact of modulating the cGMP/PKG pathway on rescuing the multiple defects of the most common and prevalent *CFTR* mutation. In particular, we investigated the effect of the cGMP-specific PDE5 inhibitor vardenafil on the accumulation and mislocalisation of the F508del-CFTR protein. We showed, at the transcriptional level, by using analogues and specific agonists of cGMP and by applying highly specific inhibitors of PKG and PKA, that epithelial bronchial cells express PDE5, an enzyme initially identified in rat platelets (Coquil et al., 1980; Hamet and Coquil, 1978) and abundantly expressed in smooth muscle cells (Moncada and Martin, 1993), including those from the pulmonary vascular bed (Francis and Corbin, 1988; Thomas et al., 1990). We confirmed that human bronchial epithelial cells display an intact cellular machinery to ensure a finely tuned balance between production and breakdown of cyclic nucleotides involved in numerous cellular signalling pathways, and that this complex machinery seemed to be preserved in CF. We analysed the interplay of specific agonists on the synthesis of the major second messengers, cGMP and cAMP. We showed that increasing cGMP intracellular contents, either by treating with vardenafil or with 8-Br-cGMP, does not interfere with the synthesis of cAMP, the best-known CFTR regulator. While these findings highlight the absence of an apparent overlap of the effect of cGMP modulators on the cAMP intracellular contents, a unidirectional cAMP-to-cGMP crosstalk was unveiled. The observation that the cAMP agonist forskolin increased cGMP contents is in line with previous reports. In fact, it has been shown that forskolin at low nanomolar concentrations stimulates cGMP accumulation in human thyroid cells (Golin-Bisello et al., 2005) independently of adenylate cyclase activation (Ho et al., 1989). At low millimolar concentrations, forskolin more selectively stimulates cAMP while the cGMP stimulation decreases (Brandi et al., 1984). The unidirectional crosstalk revealed that the cGMP intracellular signalling transduction system appears to be a multi-avenue cascade triggered by either cGMP or cAMP agonists with distinct amplitudes and kinetics of responses.

We have previously shown that *in vivo* treatment with vardenafil of F508del homozygous CF mice normalises CFTR-dependent chloride transport across the respiratory (Lubamba et al., 2008, 2011) and intestinal (Dhooghe et al., 2013) epithelia, and that it fully corrects F508del-CFTR trafficking and mislocalisation in mouse tissue sections (Dhooghe et al., 2013). Vardenafil has not been previously studied in human CF cells. The CF cell model used in this work allowed confirmation of the mistrafficking of the F508del-CFTR protein. In the present human-derived cell-based work, we observed a vardenafil-induced increase of the localisation of the mutant CFTR in the juxta-membrane areas of CF bronchial epithelial cells, even though a complete correction of the protein mistrafficking and expression at the cell surface was not achieved. Further work would be required to investigate the expression of functional rescued protein in plasma membrane. It would use fully polarised epithelial cells cultured on permeable support under immersed conditions and biochemical methods (western blot, surface biotinylation) to quantify forward CFTR trafficking and the ratio between immature and mature, or more complex-glycosylated forms of CFTR. In further functional studies, the amount of F508del protein rescued at the plasma membrane can be measured by short-circuit current (Ghanem et al., 2005) and by patch-clamp analysis (Crutzen et al., 2016) on fully polarised epithelia.

Vardenafil has not been previously tested in patients. However, clinical benefits have been obtained following treatment with its analogue, sildenafil, in the inflammatory response in patients with mild to moderate CF (Taylor-Cousar et al., 2015). The study showed that treatment with oral doses of sildenafil was safe and well tolerated. Even though under a short-term therapy over 6 weeks, 20 mg three times a day (TID) for 1 week, followed by 40 mg TID for 5 weeks, a decreased activity of sputum neutrophil elastase and a non-significant trend towards improvement in sputum IL-8 were shown (Taylor-Cousar et al., 2015). A Phase II, randomized, double-blind, placebo-controlled clinical trial (NCT01132482) assessing the effect of oral therapy of sildenafil on transepithelial sodium and chloride conductance across the nasal mucosa in 19 patients homozygous for the F508del mutation was completed in May 2017. Results of the clinical trial testing sildenafil under a shorter duration of four weeks (20 mg TID for 1 week followed by 40 mg TID for 3 weeks) are awaited. Conversely, no clinical benefit has been observed so far in a Phase II clinical trial testing the effect of escalating doses of a soluble guanylate cyclase stimulator riociguat in adult patients homozygous for the F508del mutation (Taylor-Cousar et al., 2018). In fact, an interim data analysis showed no significant change in exploratory endpoints, such as sweat chloride concentration, nasal potential difference parameters, lung clearance index and forced expiratory volume in 1 s, following 28 days of treatment with riociguat (nine patients) or placebo (seven patients). The fact that riociguat, a drug that typically activates nitric oxide to stimulate cGMP synthesis, was not confirmed to be a valid therapeutic option for CF is in line with our findings indicating that the correcting effects of vardenafil are mediated by cGMP/PKG-independent mechanisms.

To investigate the pathways of vardenafil action on the cellular contents of the F508del-CFTR protein, we looked up- and downstream in the cGMP/PKG signalling cascade by evaluating the effect of a potent, cell-permeant, highly resistant to hydrolysis, cGMP analogue and of a metabolically stable competitive PKG inhibitor. It should be kept in mind that the accumulation of cGMP is transient. The fact that the cGMP analogue alone was not able to reproduce the effects of vardenafil, and that the PKG inhibitor in combination with vardenafil was not able to prevent the observed effects, further supports the view that they are mediated by cGMP/PKG-independent mechanisms. They include biosynthetic steps of the mutant protein sweeping from early processing to translocation to juxta-membrane portions of the cells and, possibly, inhibition of proteasomal degradation. The mechanism of action of vardenafil can differ according to the cell type. We have previously shown that in immune, non-epithelial cells such as macrophages, PDE5 expression, essential for driving differentiation of the cells towards a pro-inflammatory profile, is required for the anti-inflammatory effect of vardenafil (Noel et al., 2017). Our findings are in agreement with those of (Leier et al., 2012), suggesting that the rescuing effect of sildenafil, a less selective PDE5 inhibitor having a less potent and less prolonged effect than vardenafil (Gresser and Gleiter, 2002), is independent of the cGMP/PKG pathway. In Leier's work, the effects of sildenafil assessed by transepithelial measurements conducted in the same human bronchial epithelial cells tested in this work (16HBE14o- and CFBE41o-) grown on permeable support to achieve full polarisation, were not prevented by guanylate cyclase blockers or by PKG inhibitors. The vardenafil concentration used in our work, 10 µM, was in line with that tested by Leier of 60 µM sildenafil, an equivalent vardenafil concentration (Leier et al., 2012). Our results suggest that vardenafil should be more promising than sildenafil as a therapeutic strategy for CF, since reducing active doses potentially contributes to reducing off-target effects. In addition, the duration of action of vardenafil is much longer than that of sildenafil (Gresser and Gleiter, 2002) or tadalafil (Ding et al., 2011).

A direct interaction of vardenafil with the CFTR protein leading to conformational rearrangements, as demonstrated for lumacaftor (Van Goor et al., 2011), ivacaftor (Van Goor et al., 2009; Cholon et al., 2014, 2016) and other small molecules such as VRT-325 and cor-4 (Boinot et al., 2014; Thibodeau et al., 2010; Wang et al., 2007), could be considered. The mechanism by which intracellular accumulation of F508del-CFTR by vardenafil was not followed by full membrane expression could not be addressed in this work. The fact that vardenafil mostly increased the expression of F508del in the second segment, and that this might correspond to an ER-enriched compartment could suggest that the vardenafil effect is mediated, at least in part, by increasing the availability of the F508del protein in the ER compartment (e.g. possibly via inhibition of F508del degradation). The absence of well-differentiated apical membranes in not-fully polarised bronchial epithelial cells cultured on impermeable supports (Wiszniewski et al., 2006), compared to native tissues, could, at least partly, contribute to the incomplete correction by vardenafil treatment of the mistrafficking of CFTR, an integral protein of apical membranes. However, it could be expected that in fully differentiated epithelia grown on permeable supports and under air-liquid interface conditions or even in native tissues, vardenafil alone would not be able to fully correct the multiple defects of the F508del CFTR protein. Results from clinical trials and basic studies indicate that combining compounds with different mechanisms of action is required to address the phenotypic complexity of F508del-CFTR (Keating et al., 2018). Combining different correctors, for instance an early and a late binding with a potentiator (Bell et al., 2019) has been considered a promising strategy leading to further improvements in efficacy and clinical benefits. The fact that vardenafil did not increase the amount of plasma membrane-resident CFTR may require its combination with drugs promoting protein stability in the post-Golgi compartments (Lukacs et al., 1997) and in cell membranes, or facilitating its juxta-membrane endocytic trafficking (Gentzsch et al., 2004).

Altogether, we confirmed, in human cultured bronchial epithelial cells, that vardenafil induces a rapid and transient increase in cellular cGMP contents, and that it promotes early and intermediate steps of the cellular processing of F508del-CFTR, favouring its intracellular trafficking without leading to an increased protein expression in the plasma membrane. Treatment with the PDE5 inhibitor was indeed followed by a threefold accumulation of the F508del-CFTR protein in the juxta-membrane region of the cells and a twofold accumulation in other intracytoplasmic areas. The effect on CFTR seemed to be independent of the cGMP/PKG pathway as it was not reproduced by the brominated cGMP derivative and it was not abolished by PKG inhibition. Further studies should be carried out to shed more light on the potential of vardenafil as a promising therapeutic strategy for CF to be tested in combination therapy.

## MATERIALS AND METHODS

### Cell cultures

Immortalised wild-type (16HBE14o^–^) and parental CF (CFBE41o^–^) bronchial epithelial cell lines (Ehrhardt et al., 2006; Gruenert et al., 2004) were cultured in Minimal Essential Media (Thermo Fisher Scientific) supplemented with 10% foetal bovine serum, 1% penicillin-streptomycin and 2 mM L-glutamine. Cells were cultured on impermeable support in humidified incubators at 37°C under 5% CO_2_. For the analysis of 2-ddCT, between-group comparison of the relative quantification was applied if a minimum of twofold (positive or negative) change was reached (Branford et al., 2004; Ross et al., 2006).

### RT-qPCR quantification of PDE5 transcripts in human bronchial cell lines

RNA extraction was performed using the RNeasy Mini kit (Qiagen). RNA was reverse transcribed using SuperScript^®^ VILO™ cDNA synthesis kit (Thermo Fisher Scientific) in a final volume of 20 μL. Resulting cDNA was then diluted in sterile, nuclease-free water and used as a template in subsequent RT-PCR analysis. A relative quantification of PDE5 mRNA expression was performed on an ABI 7000 RT-PCR thermocycler (Applied Biosystems) as follows: 2 min at 50°C, 10 min at 95°C, 40 cycles of 15 s at 95°C and 1 min at 60°C using SYBR Green (Thermo Fisher Scientific) in a total volume of 20 μL, as previously described (Noel et al., 2017). Each experimental condition was run in duplicate. Results represent the average of three independent experiments and were expressed as the ratio of the expression of human *PDE5* gene to the reference human housekeeping *glyceraldehyde 3-phosphate dehydrogenase* (*GAPDH*) gene.

### Measurements of intracellular cAMP and cGMP concentrations in human bronchial cell lines

16HBE14o^−^ and CFBE41o^−^ cells were grown on 10 cm^2^ dishes until reaching at least 80% of confluence. Cells were then treated for 5 min or 1 h with dimethyl sulfoxide (DMSO) as a baseline control, 10 µM vardenafil, 10 µM 8-Br-cGMP or 10 µM forskolin before being lysed for 20 min with 0.1 M HCl. All compounds were dissolved in 0.1% (v/v) DMSO. Cells were subsequently centrifuged for 10 min at 1000 ***g*** to collect supernatant. To detect low concentration of cyclic nucleotides, samples were first acetylated before being assayed by a cyclic AMP or GMP competitive enzyme immunoassay kit (SanBio) as previously described (Pradelles et al., 1989).

### Immunolabelling of CFTR protein in human bronchial cell lines

16HBE14o^−^ and CFBE41o^−^ cells grown on glass coverslips were incubated for 1 h with DMSO, 10 µM vardenafil or 10 µM 8-Br-cGMP. Treated cells were also incubated without PK inhibitor or in the presence of 1 µM PKA inhibitor (H-89 dihydrochloride hydrate, Sigma-Aldrich) or 1 µM PKG inhibitor (Rp-8-Br-PET-cGMPS, Sigma-Aldrich). Cells were fixed in ice-cold methanol for 10 min at 4°C, then blocked for 1 h with 1% bovine serum albumin in phosphate buffered saline (PBS). They were incubated overnight at 4°C with primary anti-CFTR monoclonal antibody raised against the NBD2 domain (mAb 596 diluted 1:500; obtained via the Cystic Fibrosis Foundation Therapeutics, www.cftrfolding.org/CFFTReagents.htm) and primary anti-ZO-1 (zonula occludens-1, PA5-28869; Thermo Fisher Scientific; dilution 1:200). After rinsing three times in PBS containing 0.1% Triton X-100, cells were incubated for 1 h at room temperature with goat anti-mouse (Alexa Fluor 488 IgG (H+L), 2 mg/ml; Thermo Fisher Scientific) and goat anti-rabbit (Alexa Fluor 555 IgG (H+L), 2 mg/ml; Thermo Fisher Scientific) secondary antibody, both diluted 1:1000 in PBS containing 0.1% Triton X-100. Cells were washed three times before being mounted on slides with anti-fading medium containing DAPI (4′,6-diamidino-2-phenylindole; SlowFade Gold antifade reagent with DAPI, Thermo Fisher Scientific). Specificity of the immunoreactive signal was confirmed by the generally accepted negative control consisting in omitting the CFTR primary antibody. Slides were then stored at −20°C in the dark and imaged using a Zeiss AxioImager Z1 fluorescent microscope equipped with an ApoTome module allowing structured illumination. This method allows optical sections from biological specimens with a similar or even a slightly higher lateral resolution than that achieved by laser scanning micgroscopy (Weigel et al., 2009). Optical sections were taken with a constant exposure time of 100 milliseconds using a 63× oil immersion objective. Specific staining and negative controls were photographed under identical conditions (filters, microscope magnification, and fluorescence exposure time). Morphometric analysis was performed as previously described (Dhooghe et al., 2013). Briefly, cross-sectional scans of pixel intensities were measured along a line through the widest part of the nucleus of individual cells and analysed using the AxioVision Release 4.9.1 software. The mean CFTR-related intensity across each cell (*n*=9 cells per condition, randomly selected, nuclei excluded) was measured as representative of CFTR distribution.

### Statistics

Descriptive statistics, graphs and scatterplots were performed using GraphPad Prism 8 for Windows (GraphPad Software Inc). Prior to statistical analysis, data were checked for normality of distributions (Shapiro-Wilk normality test). Between-group comparisons of parametric data were evaluated using one-way ANOVA test after checking that variances were homogeneous. Post-hoc comparisons were performed using Student's *t*-test or the Tukey–Kramer honestly significant difference test (HSD), for two or more than two x levels, respectively. Null hypothesis was rejected at *P*-values <0.05. For the analysis of 2-ddCT, between-group comparison of the relative quantification was applied if a minimum of twofold (positive or negative) change was reached (Branford et al., 2004; Ross et al., 2006).

## Supplementary Material

Supplementary information
