# Supplementary material for: Vardenafil increases intracellular accumulation of the most prevalent mutant cystic fibrosis transmembrane conductance regulator (CTFR) in human bronchial epithelial cells
Source: Biol Open. 2020 Aug 25;9(8):bio053116. doi: 10.1242/bio.053116 (PMC7473651; doi:10.1242/bio.053116)
Supplement: Supplementary information [file biolopen-9-053116-s1.pdf]

Supporting information

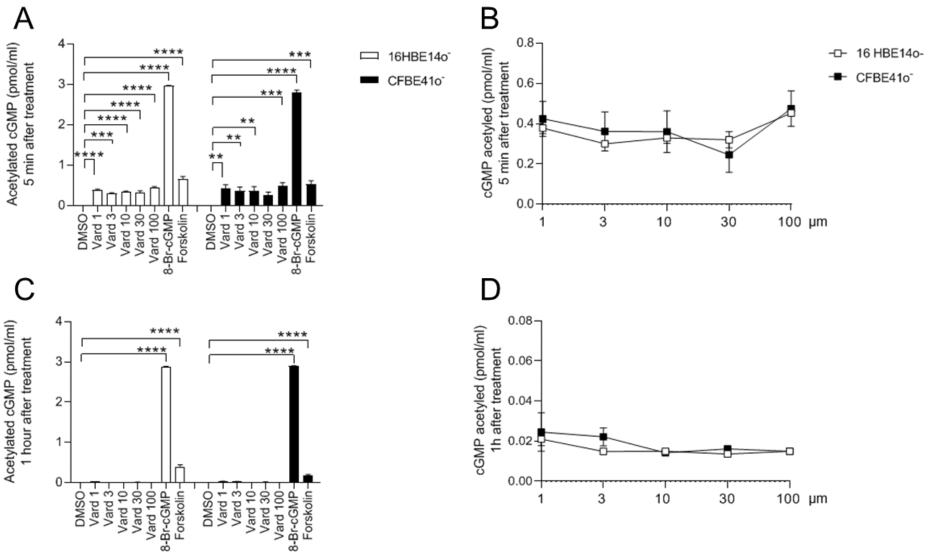

Fig. S1 Quantification of acetylated cGMP intracellular contents in 16HBE14o- cells (white bars in A,C, squares in B,D) and in CFBE41o- cells (black bars in A,B, circles in B,D) after five minutes (A,B) or one hour (C,D) of treatment with increasing concentrations of vardenafil (1, 3, 10, 30, 100 µM) or with a fixed dose (10 µM) of 8-Br-cGMP or forskolin. Data are expressed as mean ± SEM (triplicate of three individual experiments). Asterisks indicate level of significance between the control condition (DMSO) and the other groups. Comparisons were performed by ANOVA with posthoc analysis made by using Student *t* test or Tukey-Kramer Honestly Significant Difference (HSD) test, as adequate (* $p < 0.05$; ** $p < 0.01$; *** $p < 0.001$).

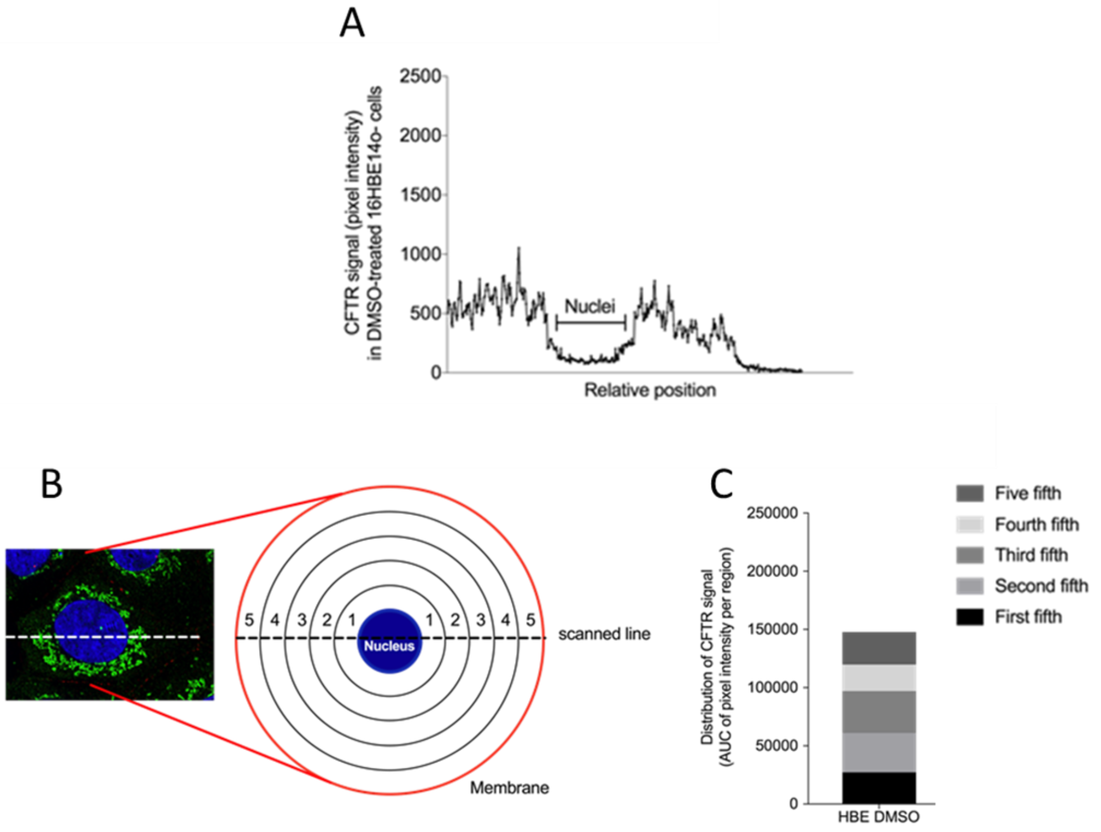

Fig. S2 Morphometric analysis of CFTR immunofluorescence of 16HBE14o- cells. Distribution of the CFTR signal intensity quantified by morphometric analysis of cross-sectional profiles of individual cells (n=9) treated with DMSO. (B) Illustration of dividing a cell scan in 5 segments from the juxta-nuclear region (1) to the membrane region (5). (C) The CFTR fluorescence signal was distributed throughout the cytoplasm of 16HBE14o- cells up to the juxta-membrane areas.

Biology Open • Supplementary information
